# Effect of antivascular endothelial growth factor treatment on the intratumoral uptake of CPT-11

**DOI:** 10.1038/sj.bjc.6601005

**Published:** 2003-06-10

**Authors:** H Wildiers, G Guetens, G De Boeck, E Verbeken, B Landuyt, W Landuyt, E A de Bruijn, A T van Oosterom

**Affiliations:** 1 Laboratory of Experimental Oncology (LEO), University Hospital Gasthuisberg, Herestraat 49, B-3000 Leuven, Belgium; 2 Department of Histopathology, University Hospital Gasthuisberg, Herestraat 49, B-3000 Leuven, Belgium; 3 Laboratory of Experimental Radiobiology/LEO; University Hospital Gasthuisberg, Herestraat 49, B-3000 Leuven, Belgium

**Keywords:** anti-VEGF mAb, CPT-11, tumour delivery, Hoechst 33342, perfusion

## Abstract

Promising preclinical activity with agents blocking the function of vascular endothelial growth factor (VEGF) has been observed in various cancer types, especially with combination therapy. However, these drugs decrease microvessel density, and it is not known whether this reduced vessel density (VD) results in decreased delivery of concomitantly administered classical anticancer drugs. We designed an *in vivo* study to investigate the relation between VEGF-blocking therapy, tumoral blood vessels, and intratumoral uptake of anticancer drugs. Nude NMRI mice bearing colon adenocarcinoma (HT29) were treated with the anti-VEGFmAb A4.6.1 or placebo. After 1 week, CPT-11 was administered 1 h prior to killing the animals. In A4.6.1 treated tumours, there was a significant decrease in VD, more pronounced with potentially functional large vessels than endothelial cords. Interestingly, a trend to increased intratumoral CPT-11 concentration was observed (*P*=0.09). In parallel, we measured an increase in tumour perfusion, as estimated by high-performance liquid chromatography determination of intratumoural Hoechst 33342 concentration. In the growth delay study, CPT-11 was at least equally effective with or without pretreatment with A4.6.1. These data suggest that tumour vascular function and tumour uptake of anticancer drugs improve with VEGF-blocking therapy, and indicate the relevance for further investigations.

Antiangiogenic therapy (AAT) inhibits new blood vessel formation and decreases vessel density (VD) and tumour growth in several tumour models (Kim *et al*, 1993; Borgstrom *et al*, 1998; Kamiya *et al*, 1999; Lee *et al*, 2000; Soh *et al*, 2000; Beecken *et al*, 2001; Shaheen *et al*, 2001). As a consequence, one would expect that tumour blood flow and tumour oxygenation also decrease during AAT. However, counterintuitive results have seemingly been demonstrated in several settings. Hypoxia is unchanged or reduced with squalamine (Teicher *et al*, 1998), the combination of TNP-470 and minocycline (Teicher *et al*, 1995b), and with the anti-VEGF mAb A4.6.1 (Lee *et al*, 2000). In addition, interstitial fluid pressure (IFP) is significantly reduced by the latter agent in the LS174T colon carcinoma model (Lee *et al*, 2000). Conclusive data concerning alterations in overall tumour perfusion after AAT are lacking. One study with interleukin-12 as an antivascular agent suggested decreased overall tumour perfusion (Gee *et al*, 2001). An interesting new concept that AAT may normalise the established tumour vasculature apart from inhibiting new vessel formation, has emerged (Jain, 2001). This ‘normalisation’ could lead to better tumour perfusion, oxygenation, and also to better delivery and efficacy of therapeutic agents. Several studies have shown that AAT potentiates the efficacy of standard anticancer drugs with enhanced delay of tumour growth (Teicher *et al*, 1992, 1994; Sweeney *et al*, 2001), stimulating interest in combining these two treatment modalities. Also, low-dose (metronomic) regimens of standard chemotherapy can have clear preclinical activity without significant toxicity, and when combined with AAT may lead to full and sustained regressions (Browder *et al*, 2000; Klement *et al*, 2000; Man *et al*, 2002). One study has suggested that pretreatment with the antiangiogenic drug TNP-470 and minocycline results in increased intratumoral drug levels of platinum (Teicher *et al*, 1995a). TNP-470 is a semisynthetic derivative of fumagillin, a naturally occurring direct inhibitor of endothelial proliferation (Ingber *et al*, 1990). Unfortunately, phase I studies with TNP-470 showed dose-limiting neurotoxicity with only minor tumour response (Bhargava *et al*, 1999; Stadler *et al*, 1999; Logothetis *et al*, 2001), and interest in using this drug clinically has diminished. Antiangiogenic therapy increases tumour radiation response, further supporting the new concept of ‘normalising tumour vasculature’ (Gorski *et al*, 1999; Lee *et al*, 2000; Kozin *et al*, 2001). In these studies, AAT decreases rather than increases hypoxia, explaining at least in part the increased radiosensitivity of the tumour cells.

Vascular endothelial growth factor (VEGF) is a key growth factor in the angiogenic process, and has a role in endothelial cell migration, proliferation, vascular permeability, and endothelial cell apoptosis. Inhibition of VEGF function by tyrosine kinase inhibitors, or monoclonal antibodies against VEGF or one of its receptors, has a significant antitumour effect. The monoclonal VEGF antibody A4.6.1 significantly decreases VD, tumour growth, and IFP, in several tumour models including colorectal cancer (Kim *et al*, 1993; Borgstrom *et al*, 1998; Lee *et al*, 2000; Rowe *et al*, 2000). This monoclonal antibody with the product name ‘Bevacizumab’ has also shown promising clinical activity without major toxicity in patients with metastatic renal cancer (Yang *et al*, 2002), and phase III clinical trials are ongoing. An increased tumour radiation response has been observed with A4.6.1 (Lee *et al*, 2000), but there are no data available regarding the effect of the antibody on the tumour availability of anticancer drugs. In this study, we have examined the effect of A4.6.1 on the intratumoral availability of the cytotoxic CPT-11 in a colorectal cancer model. Vascular function was evaluated after treatment using the perfusion marker Hoechst 33342 (H33342). CPT-11 is a topoisomerase I inhibitor that is increasingly used in colon cancer, with a biological half-life of about 1 h in mice (Kaneda *et al*, 1990). A prodrug, it is converted by decarboxylation in the liver into the active metabolite SN-38, which is much more cytotoxic than the parent compound. According to the paradigm of normalising tumour vasculature with AAT, it is possible that delivery of CPT-11 is increased rather than decreased when given with VEGF-blocking agents.

## MATERIALS AND METHODS

### Materials

The humanised murine antihuman VEGF mAb A. 4.6.1. was kindly provided by Genentech (South San Francisco, CA, USA). CPT-11 was a gift from Aventis Pharma Belgium (Brussels, Belgium). CPT-11 solution was freshly prepared in 0.9% saline at a concentration of 10 *μ*g *μ*l^−1^.

### Tumour cell lines

The HT29 human colon cancer cell line was obtained from the American Type Culture Collection (Manassas, VA, USA), and cultured under 5% CO_2_ in minimal essential medium (MEM) supplemented with 10% foetal bovine serum (FBS), 2 U ml^−1^ penicillin and streptomycin, 1 mM sodium pyruvate, 2 mM L-glutamine, and nonessential amino acids at 37%.

### Tumour xenografts in nude mice and drug administration

Athymic male NMRI nude (nu/nu) mice, 6–8 weeks old, were used. They were fed a diet of animal chow and water *ad libitum* throughout the experiment. HT29 cells (1 × 10^6^ cells in 100 *μ*l phosphate-buffered saline (PBS) were injected subcutaneously in the right and left flank of the animals. Tumour growth was assessed every 2–3 days. Three orthogonal diameters were measured with vernier callipers, and used to calculate the volume of the tumour using the formula a × b × c × *π*/6 (Tomayko and Reynolds, 1989). When unilateral or bilateral tumours reached a volume of 200–300 mm^3^, the mice were randomised into two groups for the first part of the study, examining the intratumoral CPT-11 concentration (20 tumours per group), and into four groups for the second part of the study, examining the tumour growth delay (10 tumours per group). A measure of 200 *μ*g A4.6.1, diluted in saline, was administered intraperitoneally to each mouse (day 0). The same dose was given on day 4. Control animals were injected with saline. A single injection of 100 mg kg^−1^ CPT-11 was administered intraperitoneally on day 7. This dose produces a growth delay in our tumour model (data not shown), and is known to be the LD_0_ in mice (Lavelle *et al*, 1996). Control animals were injected with saline. Body weight, tumour volume, and time between subcutaneous cell inoculation and randomisation were not different in the various groups. The time between subcutaneous cell inoculation and randomisation was between 18 and 25 days. The mean was 23.6 days (treatment group) and 23.7 days (control group) for the first part, and between 20.1 and 21.2 days for the four groups in the second part. In the second part measuring tumour growth delay, mice were sacrificed when the largest tumour exceeded 1000 mm^3^. All the animal studies are in agreement with the Guidelines for the Welfare of Animals in Experimental Neoplasia (United Kingdom Co-ordinating Committee on Cancer Research (UKCCCR) 1998), and approved by the Animal Ethics Committee of the Catholic University Leuven.

### Tissue and blood sample preparation in the first part of the study

The mice were killed at day 7 by ether inhalation one hour after the CPT-11 injection. A 500 *μ*l blood sample was taken through an intracardiac puncture and collected in an EDTA tube, and the tumours were resected. Each tumour was halved, and one half was frozen slowly in methylbutane for microscopic evaluation. The other half was snap frozen in liquid nitrogen for the later measurement of tumour CPT-11 concentrations. The blood was centrifuged at 1500 **g** for 10 min in a swing-out rotor, and 100 *μ*l supernatant plasma was removed and used to measure plasma concentrations of CPT-11 and SN-38.

### Histological analysis

Histological sections were stained for CD34 and CD105 with Biotin conjugated CD34 and CD105 antibody (BD Biosciences), at a dilution of 1 : 40, avidin–biotin complex, and diaminobenzidine as chromogen. Vessel density was counted on CD34 and CD105 immunostained slides, according to conventional stereological methods using an unbiased Gundersen counting frame (Gundersen, 1978). Briefly, 10–20 quadrats per tumour were assessed, selected via a stratified random sampling procedure at a magnification of × 200, with the aim of counting at least 50 vessels per tumour (Wildiers *et al*, 2002). The image was unfocused while moving from one quadrat to another to avoid any bias in vessel counting. Counting was performed in one section through the largest diameter of the tumour. Vessels were separated into endothelial cords, where no lumen or a lumen smaller than 7 *μ*m (the size of an erythrocyte) was observed, and into large vessels (lumen ⩾7 *μ*m). In the large vessel group, the shortest luminal diameter was measured and noted. The proportion of tumour necrosis, expressed as a percentage, was estimated at a magnification of × 40. All measurements were performed by two independent observers, with interobserver variability less than 10%, and all data were pooled for further analysis. Total VD, endothelial cord VD, and large-vessel VD were calculated.

### Determination of vascular function using the perfusion marker H33342

The use of a fluorescent dye, H33342, to visualise and quantify tumour functional vasculature when frozen sections are viewed under ultraviolet light has been described (Smith *et al*, 1988; Quinn *et al*, 1992). H33342 is frequently used as a perfusion marker in preclinical research, where the vessels with surrounding stained cells are usually counted microscopically (Thomas *et al*, 1996; Bernsen *et al*, 1999; Bussink *et al*, 2000; Ljungkvist *et al*, 2002). Although this analysis does not directly quantify the number of perfused vessels, it provides an estimate of the relative degree of perfused tumour vasculature. The results of this technique correlated with tumour blood flow measurement using doppler ultrasound in melanoma xenograft tumours (Goertz *et al*, 2002). Hoechst 33342 was freshly prepared in 1 ml physiological saline per 100 g body weight, and administered at 40 mg kg^−1^ intravenously via the tail vein. The tumours were removed 1 min after dosing. Rather than count the number of H33342-labelled cells microscopically, we decided to determine the global H33342 uptake in the tumour using HPLC. The same HPLC procedure as for the CPT-11 measurements was used (cf. *infra*).

### CPT-11 measurements in tumour tissue and plasma

A high-performance liquid chromatographic (HPLC) method was used and validated for the simultaneous determination of CPT-11, its metabolite SN-38, and H33342, in human plasma and tumour tissue. Camptothecin (CPT) was used as an internal standard. As sample pretreatment, 10 *μ*l of HPLC grade methanol was added to 50 *μ*l plasma together with 100 *μ*l internal standard solution. After vortexing, the solution was heated to 40°C for 15 min. An additional 200 *μ*l of triethylamine-acetate buffer was then added to the solution, which was centrifuged at 14 000 r.p.m. for 5 min. Finally, the supernatant was filtered over a 0.2 *μ*m PVDF HPLC-filter and 20 *μ*l was injected into the HPLC system. After mechanical mixing of tumour tissue to generate emulsions of cellular debris, a similar procedure was followed. Separation was achieved on a Waters Symmetry 300 C8 reversed-phase column (25 cm × 4.6, 5 *μ*m). The mobile phase consisted of 72% triethylamine-acetate buffer (pH 5.2) and 28% acetonitrile at a flow rate of 1.5 ml min^−1^. CPT-11 and CPT were detected by fluorescence with excitation and emission wavelengths of 369 and 424 nm, respectively. SN-38 and H33342 were detected by fluorescence with respective excitation and emission wavelengths of 376 and 534 nm. The limits of quantitation for CPT-11, SN-38, and H33342 were 0.5, 0.25 and 0.5 ng ml^−1^, respectively. Within-run and between-run precisions were less than 10% and average accuracies were between 90 and 110%.

### Statistical analysis

‘Statistica 5.5’ was used for statistical analysis. Unless indicated, Student's *t*-test was used to evaluate differences between two independent groups, and data are presented as mean±s.d. The significance level was determined at 0.05. The tumour growth delay calculations were based on the growth increase for a defined period, the tumour volume reached at a defined time point, and the time to grow from the starting volume to a higher defined volume. Since the time points of interest varied for the several questions posed, different parameters were used for the different groups.

## RESULTS

### Anti-VEGF mAb does not impair intratumoral CPT-11 delivery

**Figure 1:**
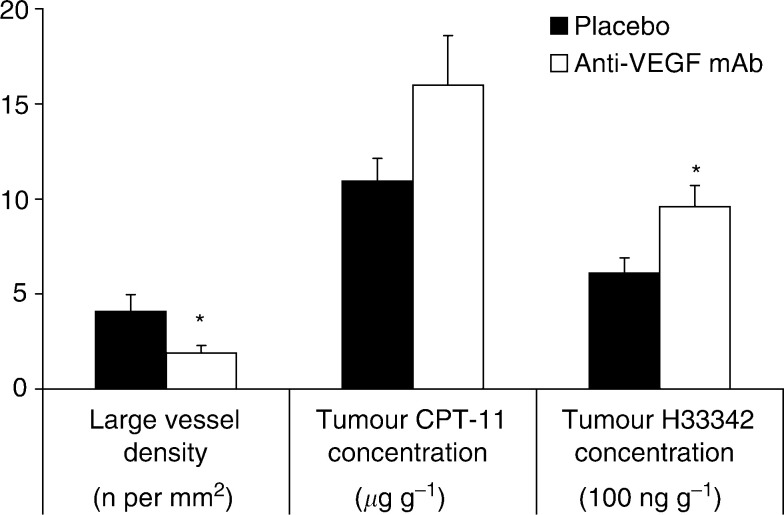
After anti-VEGF mAb therapy, large-vessel VD decreases (*P*=0.03) while intratumoral H33342, injected 1 min before killing as indicator of tumour perfusion, increases (*P*=0.01). There is a trend to higher intratumoral concentration of CPT-11 in the anti-VEGF-mAb-treated group (*P*=0.09). Asterisks, *P*<0.05 *vs* placebo. Data indicate mean±s.e.

Pretreatment with anti-VEGF mAb produces a trend to higher intratumoral availability of CPT-11; mean±s.d. 15.98± 11.67 *μ*g g^−1^ *vs* 10.93±5.39 *μ*g g^−1^ tumour tissue (*P*=0.09) (Figure 1

). There was no difference in the mean plasma CPT-11 level in the two groups; 21.31±8.03 *μ*g ml^−1^ in the anti-VEGF mAb group and 19.37±10.14 *μ*g ml^−1^ in the placebo group (*P*=0.77). The intratumoral levels of the active metabolite SN-38 were about 50 times smaller, with no significant difference between the anti-VEGF mAb group (0.26±0.10 *μ*g g^−1^) and the placebo group (0.24±0.09 *μ*g g^−1^) (*P*=0.49). The mean tumour volume at day 7, the time of excision, was significantly lower (*P*<0.001) in the anti-VEGF mAb-treated group (355±123 mm^3^) compared to the placebo group (492±113 mm^3^).

### Anti-VEGF mAb reduces tumour VD

At 1 week after the first treatment with anti-VEGF mAb, total VD decreased significantly by 20.1% on CD34 slides and 26.4% on CD105 slides (Table 1

**Table 1:** Effect of anti-VEGF mAb on tumor vessel density (VD)

	**Anti-VEGF (*n*=20)**	**Placebo (*n*=20)**	***P***	**Difference (%)**
CD34-stained sections				
Total VD (*n* per mm^2^)	36.5±3.3^a^	45.7±2.4	0.027^*^	20.1
Endothelial cords VD (*n* per mm^2^)	34.6±3.2	41.6±2.4	0.08	16.8
Large vessel VD (*n* per mm^2^)^b^	1.90±0.38	4.08±0.88	0.029^*^	53.4
Vessel diameter (*μ*m)	14.3±0.69	17.7±0.56	<0.001^*^	19.2

CD105-stained sections				
Total VD (*n* per mm^2^)	29.0±2.4	39.4±2.2	0.0026^*^	26.4
Endothelial cords VD (*n* per mm^2^)	24.9±2.4	30.8±2.3	0.078	19.1
Large vessel VD (*n* per mm^2^)	4.12±0.58	8.60±1.1	0.001^*^	52.1
Vessel diameter (*μ*m)	12.7±0.44	16.1±0.37	<0.001^*^	21.1

a Mean±s.e.

b Large vessels indicate vessels with a shortest luminal diameter larger than the size of an erythrocyte (>7 *μ*m).

**Figure 2:**
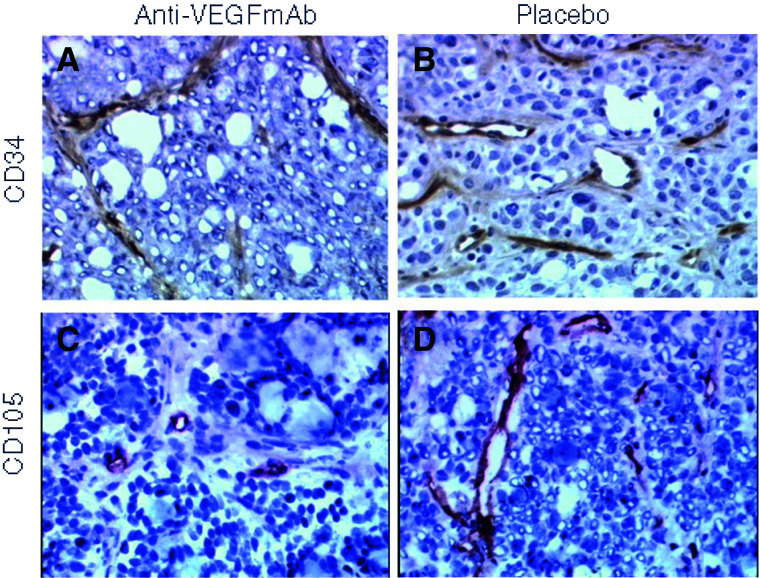
CD34 (**A, B**) and CD105 (**C, D**) staining of colorectal tumours shows decreased VD in tumours treated with anti-VEGF mAb (**A, C**) *vs* placebo (**B, D**). Original magnification × 200.

Significant at level <0.05.

). The large-vessel VD decreased by 53.4 and 52.1% on CD34 and CD105 slides, respectively. The vessel diameter of large vessels was decreased to a lesser but significant extent after anti-VEGF mAb, by 19.2 and 21.1%, respectively. Total and endothelial cord VD were lower on CD105 than on CD34 slides. In contrast, large-vessel VD was more than twice as high after CD105 compared to CD34 staining, which was confirmed by the two independent observers. There was no difference in the estimated necrosis of tumours treated with anti-VEGF mAb (37±17.6%) or placebo (34.5±21.9%) (*P*=0.69). An example of histological sections of anti-VEGF mAb and placebo-treated tumours is shown (Figure 2

).

### Tumour growth delay

**Figure 3:**
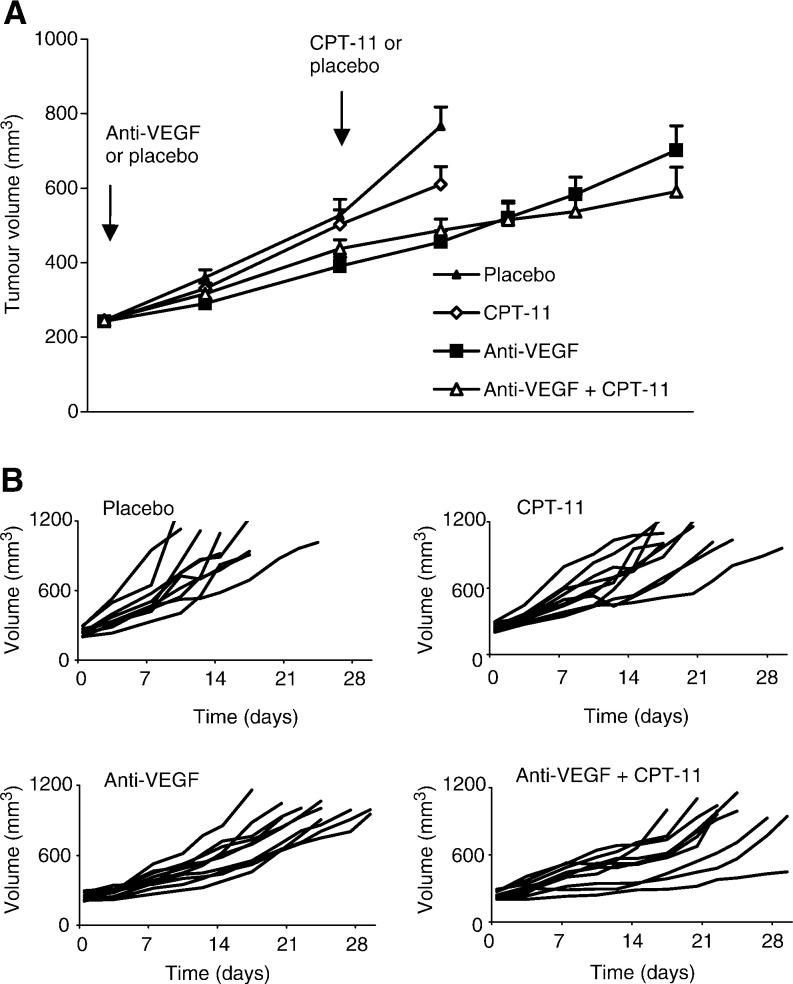
Effect of anti-VEGF mAb, CPT-11, and the combination of anti-VEGF mAb and CPT-11 on the growth of HT29 colonic tumours in mice (*n*=10 per group). (**A**) Mean tumour growth. The growth curve of each subgroup was terminated when one mouse in that subgroup developed a tumour of 1000 mm^3^, to avoid nonrepresentative mean growth curves. Data points indicate the mean±s.e. (**B**) The individual growth curves of different subgroups.

There was progressive growth of HT29 tumours up to volumes of 1000 mm^3^. Anti-VEGF mAb retarded growth significantly, with a mean tumour growth delay of 8 days. CPT-11 as a single bolus LD_0_ dose, resulted in only a moderate growth delay of about 2–3 days, which was not altered when CPT-11 was administered after pretreatment with anti-VEGF mAb (Figure 3

Table 2

**Table 2:** Effect of CPT-11 and anti-VEGFmAb on tumour growth parameter

**Effect of anti-VEGFmAb on tumour growth**	**Anti-VEGF**	**Placebo**	***P***
Start volume at day 0 (start of anti-VEGFmAb or placebo)	242±9.8^a^	244±11.2	
Growth increase from day 0 to day 10 (multiplication factor)	1.91±0.13	3.08±0.26	0.0009*
Tumour volume at day 10 (mm^3^)	458±29	766±91	0.005*
Time to grow from 250 to 900 mm^3^ (days)	21.5±1.37	13.6±1.65	0.00007*

**Effect of CPT-11 on tumour growth**	**CPT-11**	**Placebo**	***P***
Start volume at day 7 (start of CPT-11 or placebo)	502±70	527±54	
Growth increase from day 7 to day 10 (multiplication factor)	1.21±0.03	1.46±0.10	0.04*
Tumour volume at day 14 (mm^3^)^b^	660±55	840±59	0.04I
Time to grow from volume 500 to 900 mm^3^ (days)	8.73±1.28	6.61±1.04	0.12

**Effect of CPT-11 after anti-VEGFmAb on tumour growth**	**Anti-VEGF+CPT-11**	**Anti-VEGF**	***P***
Start volume at day 7 (start of CPT-11 or placebo)	407±30	391±24	
Growth increase from day 7 to day 17 (multiplication factor)	1.46±0.10	1.78±0.08	0.02*
Tumour volume at day 17 (mm^3^)	599±61	702±65	0.27
Time to grow from volume 400 to 700 mm^3^ (days)	11.35±2.9	8.76±2.28	0.04*

a Mean±s.e.

b *n*=7 in both groups.

* Significant at level <0.05.

).

### Determination of vascular function using the perfusion marker H33342

Five of the 40 tumour samples, were excluded due to analytical error. The intratumoral H33342 concentration was 57% higher in the anti-VEGF-mAb-treated group (*n*=16) than in the placebo group (*n*=19) (*P*=0.01) (Figure 1).

## DISCUSSION

### Anti-VEGF mAb does not impair intratumoral CPT-11 delivery

For any cancer treatment to be successful, it needs to meet at least two major criteria. Firstly, it must be potent and effective in destroying or inhibiting cancer cells *in vivo*, and this must be achieved with acceptable toxicity to normal host tissues. Hence, it must be possible to deliver the drug *in vivo* into the tumour tissue in sufficiently high concentrations. Different barriers to successful drug delivery have been recognised, as highlighted by Jain (1994), (1997), (1998). The chaotic blood supply, the quality of the vessel wall, and the interstitium can all play a major role in preventing efficient drug delivery (Jain, 1987). Antiangiogenic therapy can interfere significantly with these three factors, but studies of the impact of AAT on the delivery of concomitant cytotoxic drugs are very sparse.

Our investigation aimed to study this question, and to quantitatively evaluate the importance of the tumour perfusion as the first barrier. This in turn raised the question of whether VD *per se* could be a predictive tool for tumour uptake of cytotoxic drugs. Our results clearly show that anti-VEGF mAb definitely does not impair, and may even improve intratumoral uptake of CPT-11 in this tumour model. This is remarkable because anti-VEGF mAb clearly decreases tumour VD in this model. These data are consistent with our previous work where we showed a lack of correlation between melphalan tumour uptake and VD (Wildiers *et al*, 2002). A plausible explanation might be that although fewer vessels are present, they are of better quality, likely allowing improved delivery of bloodborne agents. The counting of vessels does not seem to reflect their perfusion status. Many tumour vessels are only perfused temporarily (Chaplin and Hill, 1995), or sometimes not at all (Vajkoczy *et al*, 2000). In addition to vascular length, tissue perfusion is determined by mean vessel diameter, flow resistance, and erythrocyte velocity (Intaglietta and Zweifach, 1974; Leunig *et al*, 1992; Baish *et al*, 1996; Vajkoczy *et al*, 1998). We correlated our data with measurement of functional vasculature, indicating a 57% increase in intratumoral perfusion after anti-VEGF mAb treatment, consistent with the trend of increased tumour CPT-11 uptake. Our data support the proposed concept of ‘normalising’ tumour vasculature with AAT (Jain, 2001). Pruning of immature and inefficient blood vessels by eliminating excess endothelial cells could result in a more ‘normal’ vasculature, which is better equipped to deliver nutrients and therapeutic agents. It seems plausible that the above-mentioned phenomenon is an important mechanism for the observed increased CPT-11 uptake, but other known and unknown mechanisms might be involved and are discussed below.

Apart from tumour perfusion (the ‘first’ barrier), also vascular permeability (the ‘second’ barrier) could theoretically play a role in the delivery of anticancer agents. In general, the microvasculature of solid tumours is hyperpermeable to macromolecules in comparison to normal vessels. This is presumably due to interactions between vascular endothelial cells and VEGF, also known as the vascular permeability factor (VPF). It has been demonstrated that tumour vascular permeability can be reduced by neutralization of endogenous VEGF with the anti-VEGF mAb A4.6.1 (Yuan *et al*, 1996). Although VEGF increases vascular permeability, our data do not suggest that blocking VEGF decreases permeability to CPT-11, as delivery was improved rather than diminished. Vascular permeability is probably more important for large molecules (Teicher *et al*, 1995b), and less so for small molecules (Wildiers *et al*, 2002) such as CPT-11 and SN-38, which have molecular weights of 586 and 392 g mol^−1^, respectively. In short, changes in vessel permeability do not seem to play a major role in the observed increased uptake of CPT-11.

Also interstitial transport (the ‘third’ barrier) could play a role in the intratumoral availability of small drugs such as CPT-11. The uniformly elevated interstitial fluid pressure in solid tumours leads to negligible convection in the tumour interstitium (Boucher *et al*, 1990), and drug delivery through the extracellular matrix (ECM) relies on passive diffusive transport (Netti *et al*, 1999). This transport is influenced by the constituents and characteristics of the ECM (Pluen *et al*, 2001). Interstitial pressure decreases by 74% in the LS174T colorectal cancer tumour model after pretreatment with A4.6.1 (Lee *et al*, 2000). It is therefore also possible that decreased interstitial fluid pressure allows convection again to a certain degree, leading to better delivery despite a decreased number of vessels.

Since necrosis can be expected to influence the intratumoral uptake of drugs such as CPT-11, we estimated the amount of necrosis in each tumour. Anti-VEGF mAb does not influence the amount of necrosis in this tumour type, and this aspect can therefore not explain the differences in the results we obtained on intratumoral availability of CPT-11.

Since intratumoral pH seems an important but relatively little studied parameter influencing drug uptake and resistance (Simon, 1999), pH alterations after anti-VEGF mAb could have influenced CPT-11 and SN-38 uptake. CPT-11 has a very complex pharmacology, and is known in two forms, an active lactone form and an inactive carboxylate form, between which a pH-dependent equilibrium exists that significantly impacts on the kinetic profile of the compound (Gelderblom *et al*, 1999; Mathijssen *et al*, 2001). The uptake of CPT-11 and SN-38 by intestinal cells has been demontrated to be clearly pH sensitive. At physiological pH, the uptake rates of CPT-11 and SN-38 decreased significantly by 65% at pH greater than 6.8, and accordingly, uptake rates of both lactones were significantly higher than those of their carboxylates (Kobayashi *et al*, 1999). Moreover, a decreased uptake of SN-38 with increasing pH correlated with a smaller cytotoxic effect. To our knowledge, the effect of anti-VEGF therapy on tumour pH and its correlation with uptake of anticancer drugs has not been consistently studied, and is a very interesting domain for further research.

The trend in increased intratumoral availability was only identified with the abundant parent compound CPT-11, and not for SN-38. There is no clear explanation for this at the moment; however, some remarks can be considered. Intratumoral SN-38 was only measurable at concentrations 50 times lower than CPT-11. It is known that only a limited amount of CPT-11 is transformed into SN-38 (Chabot, 1997), which agrees with plasma levels being about 12 times lower than CPT-11 in our study (data not shown). Intratumoral SN-38 levels were somewhat higher after anti-VEGF therapy, but not significantly. These results are thus not really contradictory with the CPT-11 findings.

The measurements of CPT-11 only represent concentrations in the whole tumour, not only tumour cells, but also blood vessels and the interstitium. It was not possible to distinguish differences in CPT-11 concentrations within the different compartments in the present study. Plasma and red blood cells in the tumoral vessels also contain CPT-11 (Combes *et al*, 2000), and it is therefore possible that differences in intratumoral blood volume, secondary to AAT, may be a factor. However, blood vessels comprise only about 7% of the total tumour volume in this model (from a random sample of four tumours, data not shown), and about 91% of the vessels counted were endothelial cords without a lumen (Table 1). Assuming the volume of blood in the tumour is 5% (probably an overestimate), then 20 *μ*l will be present in a tumour of 400 mm^3^ (400 *μ*l). With a plasma concentration of 20 *μ*g ml^−1^ and with approximately 40% of CPT-11 present in erythrocytes, tumoral blood only contains 0.4 *μ*g of a total tumour concentration of 4 *μ*g. Therefore, at most 10% of the intratumoral CPT-11 is derived from blood, and this amount will not greatly affect the measurements.

### Anti-VEGF mAb reduces tumour VD

As anti-VEGF mAb inhibits the formation of new blood vessels, it is logical that tumour VD decreases. However, most investigators have looked at total VD, which in some tumour types is predominantly determined by endothelial cords that presumably play only a minor role in the delivery and transport of nutrients and drugs, as there is no lumen present. We have found that VEGF mAb diminishes more the number of larger vessels than of endothelial cords in this HT29 human xenograft colorectal tumour model. This effect could be due to inhibition of larger vessel formation, or to a decrease in size of existing larger vessels. In the setting of drug delivery, and in view of transport capacity, it is important to assess the role of large vessels specifically.

To evaluate vasculature on tumour sections, various antibodies to endothelial cells (EC) are available. CD34 is one of the classical targets to identify EC, and has been called a ‘panendothelial’ marker. CD105 or endoglin is a more recently recognised endothelial antigen that is strongly expressed on proliferating EC, but little or not on resting EC. Therefore, CD105 represents a powerful marker of neovascularisation (Fonsatti *et al*, 2000, 2001). Blood vessel counts using CD105 are a better prognostic factor than CD34 in patients with breast cancer (Kumar *et al*, 1999). The vessel counts of endothelial cords, representing the majority of vessels counted, are lower with CD105 than with CD34 staining. In contrast, large-vessel VD, which reflects potentially perfused vessels, was more than twice as high with CD105 than with CD34 staining. It is known that CD34 expression on endothelial cells can be downregulated during proliferation, and endothelial cells of larger veins have been reported to be CD34 negative (Delia *et al*, 1993; Muller *et al*, 2002). Our data confirm these observations, and suggest that CD105 staining is necessary to assess large vessels.

### Tumour growth delay

The enhanced antitumour effect of chemotherapeutic agents when combined with AAT (Teicher *et al*, 1992, 1994; Sweeney *et al*, 2001) is probably not only due to differences in vascularisation, perfusion and drug delivery, but also an increase in tumour cell apoptosis (Kamiya *et al*, 1999; Wassberg *et al*, 1999). VEGF is a recognised chemoprotectant, particularly for drugs that inhibit microtubules, by reducing the proapoptotic effect of chemotherapy (Tran *et al*, 2002). Inhibiting proangiogenic factors such as VEGF with AAT may make endothelial cells more sensitive to cytotoxic agents.

The tumour growth delay with anti-VEGF mAb confirms previous observations in colon cancer and other tumour models (Lee *et al*, 2000). It must be emphasised that anti-VEGF mAb was administered only twice for the specific purpose of this study, and prolonged administration would probably result in longer growth delays. Similarly, CPT-11 was administered only as a single LD_0_ bolus. Higher response rates and increased tumour growth inhibition have been reported with fractionated regimens in mice, for example, every 4 days for 3 cycles (Lavelle *et al*, 1996), 6-day courses (O'Leary *et al*, 1999), or at low doses (10 mg kg^−1^) in protracted schedules of several weeks (Houghton *et al*, 1995), administering a higher total dose. Regardless, CPT-11 retards tumour growth with or without anti-VEGF mAb pretreatment, and our data certainly do not show antagonism between CPT-11 and anti-VEGF mAb. The anti-VEGF mAb + CPT-11 group grew faster from day 0 to day 7, in comparison with the anti-VEGF mAb alone group (Figure 3), but not significantly (*P*=0.68). Nevertheless, following CPT-11 administration to the former group at day 7, the growth curves clearly cross, indicating effective growth inhibition in this subgroup.

It is also interesting that a clear antiangiogenic effect of CPT-11, separate from the antitumour cell effect, has been reported (O'Leary *et al*, 1999). The study period allowing only 1 h exposure to CPT-11 was too short to evaluate this effect.

It is remarkable that the anti-VEGF mAb A4.6.1 substantially reduces, but does not completely suppress, tumour growth after systemic administration. The main mechanism of action is probably blockade of human VEGF (VEGF 165) in the xenograft tumours. However, it has been shown that host VEGF (VEGF 164 in mice) can also significantly contribute to tumour growth (Gerber *et al*, 2000). For maximum inhibition of tumour growth in human xenograft tumour models in mice, it is probably necessary to block VEGF completely.

### Determination of vascular function using the perfusion marker H33342

H33342 is used as an index of functional vascular volume, and quantified with a random sampling procedure using the Chalkley method on histological slides (Smith *et al*, 1988; Quinn *et al*, 1992). However, the detection of all the H33342 in the tumour may be a more global index of whole tumour perfusion. Using the same HPLC conditions as for CPT-11 and SN-38, we were able to detect and quantify H33342 as a distinct peak on the chromatogram. This concentration reflects the global uptake of H33342, but can be altered by many physiological parameters including blood flow, permeability, blood pressure, and interstitial pressure. It is a recognised relative rather than an absolute index of perfusion and functional vasculature, allowing comparison between different treatments in tumour models. This approach is original, objective, and feasible when HPLC technology is available. H33342 is vasoactive at doses above 10 mg kg^−1^ (Trotter *et al*, 1990). However, the validity of the data is not affected, as such an effect would only be expected to influence absolute, but not relative, uptake. The uptake in treated tumours was compared to that of controls.

In summary, our results show that the anti-VEGF mAb tends to improve CPT-11 delivery, and this corresponds to an increase in tumour perfusion, even though VD is significantly decreased. This increased perfusion is a plausible cause of the observed increase in CPT-11 uptake, although other phenomena might be involved. Interestingly, the tumour growth delay induced by the combination of anti-VEGF mAb and CPT-11 is at least additive. Tumour VD lacks predictive power for intratumoral delivery of anticancer drugs. Immunohistological staining of endothelial cells with CD34 and CD105, and the separation of endothelial cord and large vessel densities, provide additional information on tumour vascularisation. A new index of functional vasculature and perfusion using HPLC determination of intratumoral H33342 is described, allowing comparison of different treatments in tumour models.
